# Gold(III) Complexes with Aromatic Cyano-Substituted Bisdithiolate Ligands as Potential Anticancer and Antimicrobial Agents

**DOI:** 10.3390/molecules30153270

**Published:** 2025-08-04

**Authors:** Dulce Belo, Sandra Rabaça, Sara G. Fava, Sílvia A. Sousa, Diogo Coelho, Jorge H. Leitão, Teresa Pinheiro, Célia Fernandes, Fernanda Marques

**Affiliations:** 1 Centro de Ciências e Tecnologias Nucleares, Instituto Superior Técnico, Universidade de Lisboa, Estrada Nacional 10, Km 139.7, Bobadela, 2695-066 Loures, Portugal; sandrar@ctn.tecnico.ulisboa.pt (S.R.); sara.f.goncalves@tecnico.ulisboa.pt (S.G.F.); celiaf@ctn.tecnico.ulisboa.pt (C.F.); 2 Departamento de Engenharia e Ciências Nucleares, Instituto Superior Técnico, Universidade de Lisboa, Estrada Nacional 10, Km 139.7, Bobadela, 2695-066 Loures, Portugal; murmur@ctn.tecnico.ulisboa.pt; 3 iBB-Institute for Bioengineering and Biosciences, Associate Laboratory i4HB—Institute for Health and Bioeconomy, Instituto Superior Técnico, Universidade de Lisboa, Av. Rovisco Pais, 1049-001 Lisboa, Portugal; sousasilvia@tecnico.ulisboa.pt (S.A.S.); diogocoelho@tecnico.ulisboa.pt (D.C.); jorgeleitao@tecnico.ulisboa.pt (J.H.L.); 4 Department of Bioengineering, Instituto Superior Técnico, Universidade de Lisboa, 1049-001 Lisboa, Portugal

**Keywords:** gold complexes, gold dithiolates, anticancer, antimicrobial, mechanism of action, structure activity relationship

## Abstract

Cancer and infectious diseases are major causes of global morbidity and mortality stressing the need to find novel drugs with promising dual anticancer and antimicrobial efficacy. Gold complexes have been studied for the past years due to their anticancer properties, with a few of them displaying antimicrobial properties, which support their pharmacological interest. Within this scope, we investigated six gold bisdithiolate complexes [Au (bdt)_2_]^−^ (**1**), [Au (dcbdt)_2_]^−^ (**2**), [Au (3-cbdt)_2_]^−^ (**3**), [Au (4-cbdt)_2_]^−^ (**4**), [Au (pdt)_2_]^−^ (**5**) and [Au (dcdmp)_2_]^−^ (**6**), and) against the ovarian cancer cell lines A2780 and A2780cisR, the Gram-positive bacteria *Staphylococcus aureus* Newman, the Gram-negative bacteria *Escherichia coli* ATCC25922 and *Burkholderia contaminans* IST408, and the pathogenic yeasts *Candida glabrata* CBS138 and *Candida albicans* SC5134. Complexes **2** and **6**, with ligands containing aromatic pyrazine or phenyl rings, substituted with two cyanonitrile groups, showed after 24 h of incubation high anticancer activities against A2780 ovarian cancer cells (IC_50_~5 µM), being also able to overcome cisplatin resistance in A2780cisR cells. Both complexes induced the formation of ROS, activated caspase-3/7, and induced necrosis (LDH release) in a dose-dependent way, in a greater extent in the case of **6**. Among the bacterial and fungal strains tested, only complex **6** presented antimicrobial activity against *S. aureus* Newman, indicating that this complex is a potential novel anticancer and antibacterial agent. These results delve into the structure-activity relationship of the complexes, considering molecular alterations such as replacing a phenyl group for a pyrazine group, and the inclusion of one or two cyanonitrile appendage groups, and their effects on biological activity. Overall, both complexes were found to be promising leads for the development of future anticancer drugs against low sensitive or cisplatin resistant tumors.

## 1. Introduction

Cancer and infection represent the major global health burden of diseases and one of the leading causes of death worldwide [1]. A number of infectious agents have been identified which cause or contribute to specific human cancers, and the other way round, specific cancers are known to be associated with infectious pathogens [2,3,4]. The mechanisms of the association infection-cancer include immune response impairment, chronic inflammation that lead to continued cell proliferation and increased risk of oncogenic transformation [5,6].

For patients with bacterial infectious diseases, resistance against commercially available antibiotics is becoming more common, and leading to untreatable infections [7]. Although several novel approaches have been proposed to treat these microbial infections, no new antimicrobials have been produced recently. For patients with cancer diseases, the high mortality rate is attributed mostly to drug resistance, genetic factors and DNA impairment ability [8,9]. Common features of antimicrobial and anticancer drugs, such as cell targets, mechanisms of action and similar mechanisms of resistance have stressed the development of novel alternatives with broad spectrum activity, presenting dual antimicrobial and anticancer properties [10,11,12].

Under the scope of drug discovery and development, metal complexes have become an emerging tool being widely used as therapeutic options to treat several human diseases. Moreover, they also present many advantages over the conventional organic compounds [13]. Incorporation of metal ions into rational ligands offers a high versatile scaffold to build a variety of distinct molecular structures, conferring a wide spectrum of coordination numbers and geometries, as well as kinetic properties [14,15,16,17,18].

Cisplatin, the first and most cited anticancer platinum metal complex, has been used for the treatment of prostate cancer, ovarian cancer, bladder cancer and lung cancer. Its mode of action mainly relies on the inducing of apoptosis and DNA damage. Although effective for the treatment of solid tumors, cisplatin induces severe side effects associated with drug resistance and tumor relapsing [12,19,20,21,22]. Complexes of transition metals other than platinum can exhibit anticancer activity with less adverse effects and different mechanism of action. Towards this goal, new strategies have been explored aiming to design anticancer metal complexes that can exhibit modes of action distinct from those of cisplatin. The goal is to hit unique biomolecular targets, ideally to reduce systemic toxicity, and widen the range of treatable cancers [23,24]. Although several complexes have shown promising anticancer activity after being tested in vitro, only a few were tested in vivo or even progressed to clinical trials [25].

Gold complexes have been considered potential alternatives to overcome resistance, as they present a different mode of action compared to cisplatin. However, the suitability of gold complexes for medicinal use is dependent on the coordinating ligands that are crucial to forming stable compounds. In this direction, several gold(I) complexes coordinated to phosphines, N-heterocyclic carbenes (NHCs), and thiosemicarbazones have emerged with promising biological properties [26,27,28]. Among them, auranofin, a thiolate-phosphine gold(I) complex, initially used to treat rheumatoid arthritis, has been repurposed as anticancer agent presenting also antimicrobial activity, even though the underlying mechanisms still remain unclear [29,30].

Aiming at the development of gold(III) complexes, efforts have been made to prevent gold(III) reduction under physiological conditions using nitrogen donors, dithiocarbamate and C^N cyclometalled ligands [31,32]. This type of ligands can provide to the Au^3+^ centers enough stability under physiological conditions. According to several reports, these complexes can effectively overcome cisplatin-resistant cancer cells, which suggests that the mechanism of action probably differs from that of cisplatin. Moreover, they can also target important antioxidant pathways such as the two cellular disulfide reductase systems glutathione (GSH) and thioredoxin (Trx) [33,34,35].

**Figure 1:**
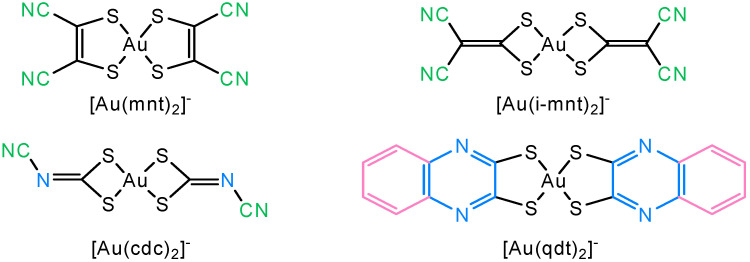
Structural formulas of the monoanionic gold complexes [Au(mnt)_2_]^−^, [Au(i-mnt)_2_]^−^, [Au (cdc)_2_]^−^ and [Au(qdt)_2_]^−^, (where mnt = 1,1-dicyanoethylene-2,2′-dithiolate, i-mnt = 2,2-dicyanoethylene-1,1-dithiolate, cdc = cyanodithioimido carbonate) and qdt = quinoxaline-2,3-dithiolate).

**Figure 2:**
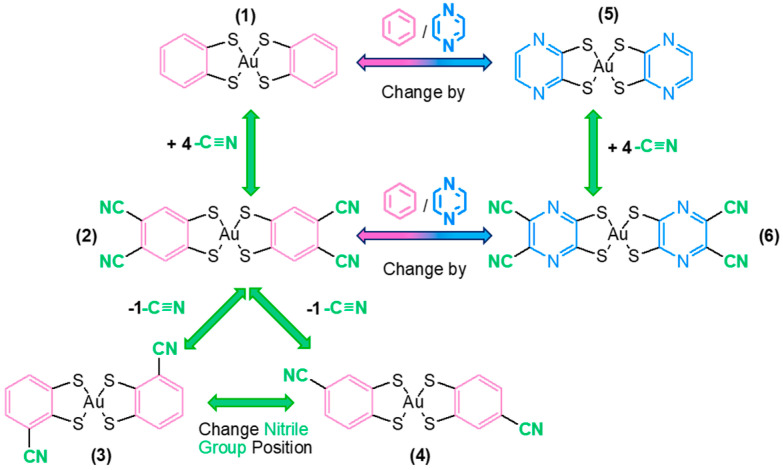
Structural formulas and structural relationship between the gold monoanionic complexes under study: [Au(bdt)_2_]^−^ (**1**), [Au(dcbdt)_2_]^−^ (**2**), [Au(3-cbdt)_2_]^−^ (**3**), [Au(4-cbdt)_2_]^−^ (**4**), [Au(pdt)_2_]^−^ (**5**) and [Au(dcdmp)_2_]^−^ (**6**).

Gold(III) complexes have been investigated mainly as anticancer agents; however, their antimicrobial properties have not been much explored. Therefore, and driven by the utmost importance of finding alternatives to fight against cancer and microbial infections, some reports showed that gold(III) bisdithiolate complexes possess these combined anticancer and antimicrobial properties [36,37,38]. These findings prompt us to investigate novel gold(III) bisdithiolate complexes exploring structural features, envisaging their potential use to treat cancer and infectious diseases. Towards this goal and taking as a starting point results obtained with ligands containing cyanonitrile groups or aromatic rings (pyrazine and phenyl) in their molecular structures (Figure 1) [36], a more systematic study evaluating the relative importance of each particular functional group was performed herein (Figure 2). The inclusion of the cyano-nitrile group and its systematic investigation in our study were motivated by this preliminary screening results [36], which suggested that such functional groups may play a relevant role when incorporated into dithiolate-based systems. It is also know that, in anti-inflammatory and anticancer drugs, nitrile groups enhance binding affinity through hydrogen bonding as well as π–π interactions and improve pharmacokinetics by increasing metabolic stability. Modifying the position and substitution pattern of the nitrile can dramatically influence potency and selectivity, making it a key tool in drug design [39].

Six gold complexes, [Au(bdt)_2_]^−^ (**1**), [Au(dcbdt)_2_]^−^ (**2**), [Au(3-cbdt)_2_]^−^ (**3**), [Au(4-cbdt)_2_]^−^ (**4**), [Au(pdt)_2_]^−^ (**5**) and [Au(dcdmp)_2_]^−^ (**6**), were assessed against ovarian cancer cells A2780 and A2780cisR, the Gram-positive *Staphylococcus aureus* and the fungal species *Candida glabrata* (bdt = benzene-1,2-dithiolate, dcbdt = 4,5-dicyanobenzene-1,2-dithiolate, 3-cbdt = 3-cyanobenzene-1,2-dithiolate, 4-cbdt = 4-cyanobenzene-1,2-dithiolate, pdt = pyrazine-1,2-dithiolate and dcdmp = 4,5-dicyanopyrazine-1,2-dithiolate). The results of these studies help to elucidate structure-activity relationships, considering the alterations in the drug molecule such as the change in a phenyl group for a pyrazine group, and the inclusion or not of one or two cyanonitrile appendage groups, and their effects on the complexes biological activity.

## 2. Results and Discussion

### 2.1. Syntheses and Characterization

**Scheme 1:**
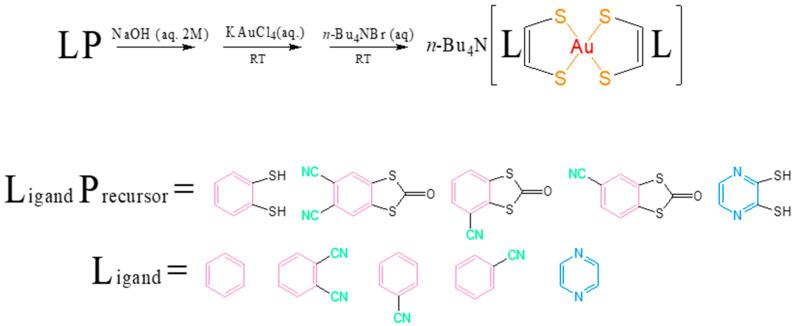
Synthetic pathway using water as the only solvent for the preparation of the tetrabutylammonium salts of [Au(bdt)_2_]^−^ (**1**), [Au(dcbdt)_2_]^−^ (**2**), [Au(3-cbdt)_2_]^−^ (**3**), [Au(4-cbdt)_2_]^−^ (**4**) and [Au(pdt)_2_^−^ (**5**). Compound **6** was prepared as in Ref. [43].

Although the synthesis of **1**–**6** can be found in the literature [40,41,42,43,44,45], in this work, except in the case of **6**, syntheses were carried out using only water as a solvent, eliminating the need to use aqueous solutions with ethanol or other organic solvents, making the whole process greener and more sustainable (Scheme 1). The synthesis of compound **6** using only water as the solvent resulted in little to no product formation, likely due to the instability of the precursors in this medium. In all cases, the procedure and yield were similar to the obtained in the syntheses previously reported [40,41,42,43,44,45]. All compounds were characterized by elemental analysis, HPLC and mass spectroscopy. Since the complexes were obtained as single crystals (except for **4**), their identity was also confirmed, by comparison with the reported unit cells, by X-ray diffraction studies.

The synthesis consisted of a hydrolytic cleavage of the ketones, in the case of **2**, **3** and **4**, or the deprotonation of the dithiol, for **1** and **5**, with a 2 M NaOH aqueous solution, followed by successive additions of KAuCl_4_ and tetrabuthylamonium bromide. In all cases, the reaction afforded a greenish polycrystalline sample, which was recrystallized from acetone/isopropanol (3:1) to give green single crystal of the complexes.

Although none of the redox potentials listed in Table 1 were determined in the course of this work, they have been compiled from the literature for clarity and comparison purposes [40,41,42,43,44,45]. Understanding the redox behavior of these complexes may be essential, as their redox potentials can play a critical role in determining their mechanism of action in biological environments. Compound **6** stands out due to its facility to reduce to the dianionic species, remaining stable over a wide potential window [−0.6 V, 1.5 V]. Compound **2** also shows a distinct behavior, as it is the only complex that undergoes a partial oxidation step prior to reaching the neutral species. The differences observed underscore the impact of ligand on the electrochemical properties of these family of complexes.

Recently, a study that quantitatively assessed the nature and strength of Au–dithiolate bonds in monoanionic gold bis (1,2-dithiolate) homoleptic complexes concluded that Au–dithiolate interactions are predominantly electrostatic, with limited covalent character [46]. It also showed that ligand substituents significantly influence the bond strength and the overall stability of the complexes, with electron-withdrawing groups such as cyano moieties leading to reduced stability. This effect is attributed to the extended π-conjugation in substituted dithiolate ligands, which can delocalize electron density away from the metal center. While no direct measurement of gold–ligand bond strength was performed in the present work, this literature insight may be relevant to understanding potential differences in reactivity or mechanism of action in biological environments. Complexes bearing CN-substituted aromatic ligands, such as [Au(dcbdt)_2_]^−^ (**2**), might be more prone to gold release under physiological conditions compared to those with non-substituted ligands, such as [Au(bdt)_2_]^−^ (**1**).

#### 2.1.1. Hirshfeld Surface Analysis and Fingerprint Plots

Hirshfeld surface (HS) analysis was employed to explore the intermolecular interactions present in the crystal structures. These surfaces are generated based on electron density distributions, approximated as the sum of spherical atomic densities of the constituent atoms. Each HS is specific to the crystal structure and reflects its unique atomic environment. In this study, HS analyses were performed for compounds **1**–**3**, **5** and **6** using CrystalExplorer 17 [47]. In the case of **4**, no good quality crystals could be obtained to determine the crystal structure.

The normalized contact distance ($d_{norm}$), which incorporates both $d_{e}$ (distance from a surface point to the nearest external nucleus) and d_i_ (distance to the nearest internal nucleus), together with the van der Waals radii of the atoms, as defined in Equation (1), serves to highlight regions of significant intermolecular interactions. The HS mapped over $d_{norm}$ values provides a visual tool to identify and interpret these contacts.

(1) $$d_{norm}=\frac{d_{i}-r_{i}^{vdW}}{r_{i}^{vdW}}+\frac{d_{e}-r_{e}^{vdW}}{r_{e}^{vdW}}$$

**Figure 3:**
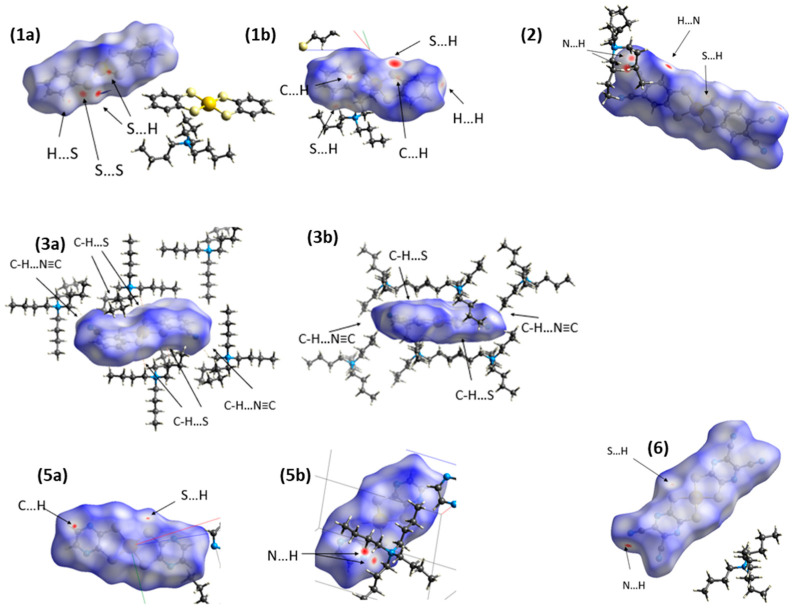
Hirshfeld surfaces of compounds **1**–**3**, **5** and **6** mapped over $d_{norm}$: [Au(bdt)_2_]^−^ ((**1a**) and (**1b**)); [Au(dcbdt)_2_]^−^ (**2**); [Au(3-cbdt)_2_]^−^ ((**3a**) and (**3b**)); [Au(pdt)_2_]^−^ ((**5a**) and (**5b**)) and [Au(dcdmp)_2_]^−^ (**6**).

Figure 3 presents the Hirshfeld surfaces of the anionic components in compounds **1**, **2**, **3**, **5** and **6**, coloured according to $d_{norm}$ values within the range −0.5383 to 1.3409. The colour scale reflects the nature and intensity of intermolecular contacts: intense red spots indicate close contacts (shorter than the sum of van der Waals radii), white regions correspond to contacts near van der Waals distances, and blue areas represent longer separations.

The red spots observed across all surfaces are indicative of strong directional interactions, notably H···N, H···S, and H···C contacts. These arise from interactions between the anionic gold bisdithiolene complexes and the cationic tetrabutylammonium moieties, including C–H···N≡C (benzocyano), C–H···N (pyrazine), C–H···S, and C–H···C contacts.

**Figure 4:**
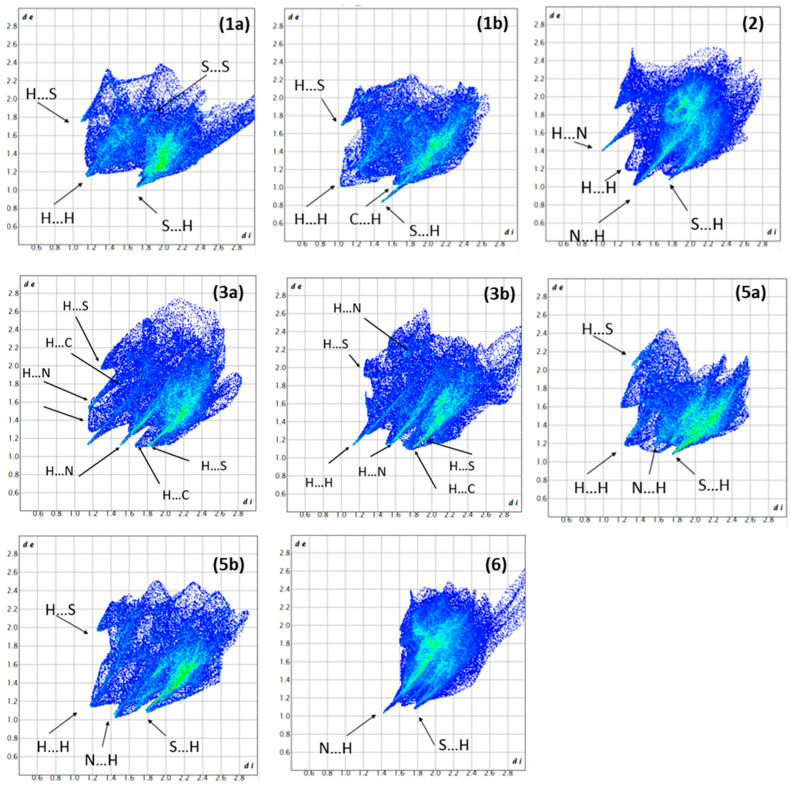
Two-dimensional fingerprint plots mapped over all molecular interaction of [Au(bdt)_2_]^−^ ((**1a**) and (**1b**)); [Au(dcbdt)_2_]^−^ (**2**); [Au(3-cbdt)_2_]^−^ ((**3a**) and (**3b**)); [Au(pdt)_2_]^−^ ((**5a**) and (**5b**)) and [Au(dcdmp)_2_]^−^ (**6**).

In addition, 2D fingerprint plots (Figure 4), derived from the combination of $d_{e}$ and $d_{i}$, illustrate the relative contribution of various types of intermolecular contacts across the different structures. These plots complement the HS analysis by quantifying the interactions.

**Figure 5:**
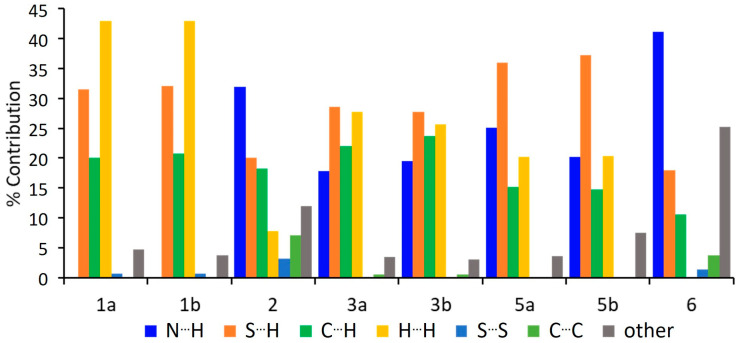
Percentage contributions of the various contacts to the Hirshfeld surface for compounds **1** (a,b), **2**, **3** (a,b), **5** (a,b) and **6**.

A detailed examination of the percentage contributions derived from the Hirshfeld surface analysis is presented in Figure 5. The results reveal that interactions involving N···H, S···H, C···H and H···H are predominant across the studied compounds. In contrast, S···S and C···C contacts contribute minimally. Notably, S···S interactions are entirely absent in compounds **3** and **5**, while C···C interactions are not detected in compounds **1** and **5**.

Although this type of analysis cannot be performed in solution, the Hirshfeld surface analysis in the solid state offers valuable insight into the potential interaction patterns that these molecules can exhibit. It clearly reveals the different types of intermolecular contacts that the anions can establish with surrounding species, serving as a representative example of the diverse interaction possibilities provided by these compounds. These variations are largely governed by the nature of the ligand in the bisdithiolate complexes, which modulates both the geometry and the interaction landscape of the resulting molecular assemblies.

#### 2.1.2. Studies on Complexes Stability in Solution by UV–Vis Spectroscopy

**Figure 6:**
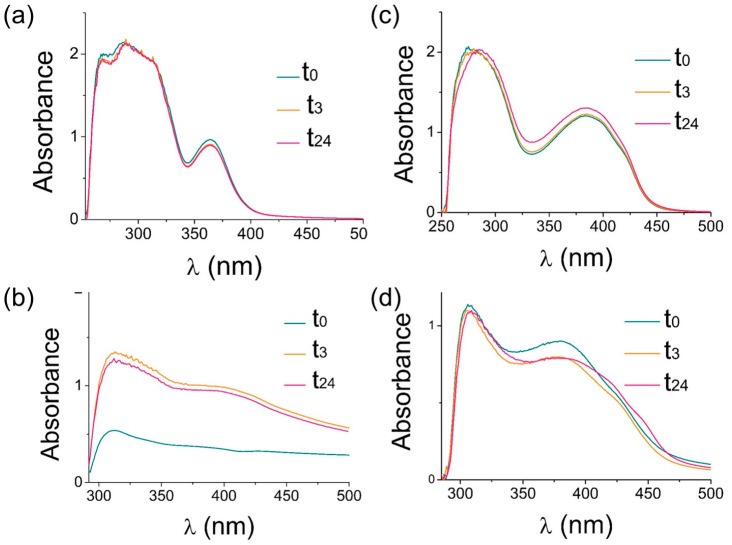
UV–Vis spectra of complex **2** (**a**) and **6** (**c**) in DMSO solution, and in phenol red-free DMEM/F12 medium in the presence of FBS (**b**) and (**d**), for **2** and **6**, respectively, at 0 h (t0, green), 3 h (t3, yellow), and 24 h (t24, pink).

The stability of the compounds in solution was assessed by UV-Vis spectroscopy at 37 °C over a 24 h period, with measurements recorded at 0, 3, and 24 h. In all cases, spectra were acquired from solutions prepared in 89% phenol red-free DMEM/F12 medium supplemented with 10% fetal bovine serum (FBS) and 1% DMSO containing the respective compound. For comparison, reference spectra of complexes **1**–**6** in pure DMSO were also collected. Figure 6 illustrates the results obtained for compounds **2** and **6**; the remaining data are provided in the Supplementary Materials. For compound **2**, the absorbance at 24 h is lower than that observed at earlier time points. This may be attributed to a gradual improvement of the compound solubility over time at 37 °C. All DMSO solutions remained translucent over the entire 24 h period, with no visible signs of precipitation. In contrast, among the solutions prepared in DMEM/FBS medium, only compound **5** remained fully translucent. Compounds **3** and **6** appeared cloudy, while visible precipitation was observed in the case of compounds **1**, **2**, and **4**. In all cases, the spectral profiles in DMEM/FBS medium differed from those in pure DMSO, generally exhibiting red-shifted absorbance peaks. This shift is likely due to interactions between the compounds and components of the medium. None of the UV-Vis spectra showed any significant changes over the monitored period, indicating that the compounds remained stable under the experimental conditions. It is worth noting that the concentrations used in these stability assays were considerably higher than those employed in cellular cytotoxicity assays, where no precipitation was observed. Further dilution of the solutions was avoided to ensure that absorbance values remained within the detection range of the spectrophotometer.

### 2.2. Biological Studies

#### 2.2.1. Anticancer Activity Assessment

The anticancer activity of the complexes was evaluated in the ovarian cancer cell lines A2780 and A2780cisR, respectively, sensitive and resistant to cisplatin. Cells were treated with complexes **1**–**6** at concentrations in the range 0.01–100 μM at several incubation times up to 48 h. The IC_50_ values were calculated from dose–response curves using the colorimetric MTT assay. Our results show that the compounds cytotoxic profile strongly depends on the presence and number of cyanonitrile groups. Upon 24 h incubation, **2** and **6** were the most active complexes, displaying a similar cytotoxic activity of about 5 µM. Notably, in the A2780cisR cells at 24 h treatment both **2** and **6** displayed similar high activity far superior than cisplatin (45 µM), which revealed that they were able to overcome resistance (Table 2). The gold salt KAuCl_4_, the counter-ion tetrabutylammonium and the thiol ligand assayed at 24 h incubation with the A2780 cells were without cytotoxic effect (IC_50_ > 100 µM).

A structure–activity relationship (SAR) was evident at 48 h, with cyanonitrile substitution playing a key role in modulating anticancer activity. Compounds **1** and **5**, which lack cyanonitrile groups, showed the lowest activity. In contrast, compound **2**, featuring a phenyl ring doubly substituted with cyanonitrile groups, was the most active, suggesting a strong contribution of electron-withdrawing substituents to the observed effect. Compound **3**, bearing a single cyanonitrile at the 3-position, also showed high activity, closely followed by compound 6 (with a pyrazine ring with two cyanonitrile groups) and compound **4**, the 4-substituted isomer of compound **3**, indicating that both the number and position of the cyanonitrile groups influence activity. The presence of a phenyl group appears to be more favorable for anticancer activity than that of a pyrazine ring.

When compared to the monoanionic gold complexes reported in Reference [36], compound **2** outperformed the best result obtained for *n*-Bu_4_N[Au(cdc)_2_], a gold complex comprising a cyanonitrile carbonate moiety. Compound **3**, **4** and **6** showed slightly lower activity than *n*-Bu_4_N[Au(qdt)_2_], a complex bearing a quinoxaline unit (a fused phenyl–pyrazine system), but a comparable activity to the simpler gold complex containing two cyanonitrile-substituted i-cyanoethylene units, *n*-Bu_4_N[Au(mnt)_2_].

Notably, compound **6**—structurally related to *n*-Bu_4_N[Au(qdt)_2_], but with the phenyl unit replaced by two cyanonitrile groups—displays slightly lower activity, yet remains within the same order of magnitude, underscoring the critical role of extended π-delocalization in enhancing biological performance.

#### 2.2.2. Cellular Uptake

**Figure 7:**
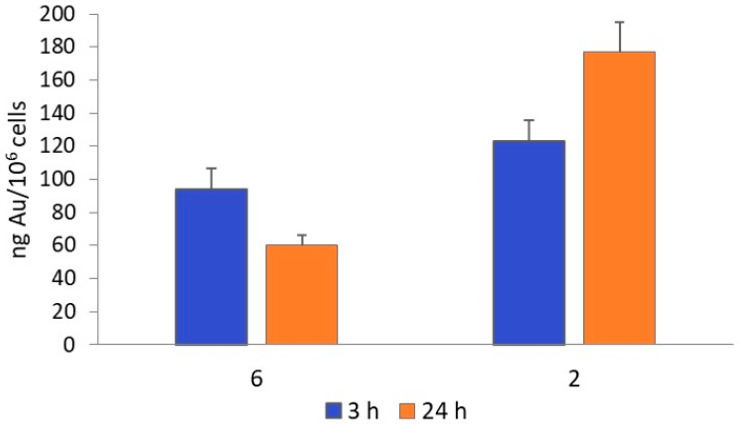
ICP-MS analysis of the cellular content of gold in the A2780 cells upon exposure to [Au(dcbdt)_2_]^−^ (**2**) and [Au(dcdmp)_2_]^−^ (**6**) at 3 h and 24 h at their IC_50_ values for each incubation time. Results (ng Au/10^6^ cells) are the mean ± SD of two independent experiments.

The cellular uptake of complexes **2** and **6** by the A2780 cells was assessed by PIXE after 3 h incubation at their IC_50_ values (80 and 20 μM, respectively), and by ICP-MS after 3 h and 24 h at their IC_50_ values (5 μM, 24 h) (Table 2) by measuring the total gold content (ng Au/10^6^ cells) in the bulk A2780 cell pellets. The complexes selection was mainly based on their cytotoxic activity profile over time and their ability to overcome resistance in the A2780cisR cells. Since both complexes are very cytotoxic upon 24 h incubation it was not possible to measure the amount of gold by PIXE at this incubation time as the concentration in medium is low and the measurement of gold by PIXE approaches the minimum detection limit. Nevertheless, concentrations obtained at 3 h with PIXE in total cellular extracts (146 ± 5.4 and 51 ± 4.6 ng Au/10^6^ cells for **2** and **6**, respectively) match in amount those obtained with ICP-MS (Figure 7), in particular for complex **2**, which strengthens the reliability of the measures, providing a quantitative approach to the amount of compound internalized in the cells. As can be observed from Figure 7, the uptake of complex **6** in terms of gold decreased over time, while for complex **2** an increase over the incubation time was observed. The amount of gold measured by PIXE in total cellular extracts after 6 h incubation at 10 μM showed that the uptake rate of **2** and **6** follow the same trend, although lower for **6** compared to **2** (344 ± 63 and 132 ± 16 ng Au/10^6^ cells for **2** and **6**, respectively).

Lipophilicity plays a crucial role in the pharmacokinetics and pharmacodynamics of a drug substance affecting its ability to cross biological membranes by passive diffusion and reach target tissues. Therefore, lipophilicity is considered a key parameter for predicting the biological activity of potential drugs and can be described as the logarithmic *n*-octanol-water partition coefficient (Log Po/w). The lipophilicity for complexes **6** (Log Po/w = 1.03) and **2** (Log Po/w = 3.11) was determined in silico using the SwissADME Web tool (login-free website: http://www.swissadme.ch, accessed on 3 June 2025) that uses multiple predictors to generate consensus estimation and increase accuracy [48]. In fact, the higher lipophilicity found for complex **2** may explain the superior cellular uptake found for this complex in comparison to complex **6**. However, an increase in the lipophilicity and cellular uptake of **2** did not result in enhanced anticancer activity, although further results are necessary to support this observation.

#### 2.2.3. Production of Intracellular ROS

**Figure 8:**
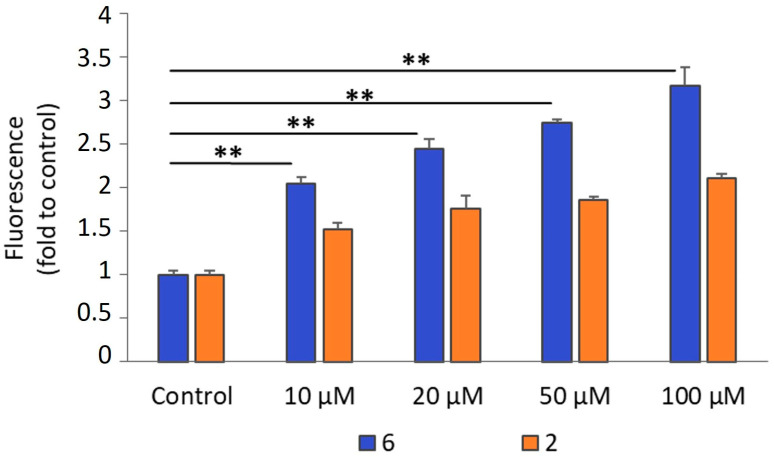
ROS production (namely peroxides) by the A2780 cells using the H_2_DCFDA probe. Cells were treated with the complexes [Au(dcbdt)_2_]^−^ (**2**) and [Au(dcdmp)_2_]^−^ (**6**) at 10, 20, 50, and 100 μM for 1 h. Results in fluorescence (fold to control) are mean ± SD of at least 2 experiments with 3–4 replicates per condition. Statistics shown for complex **6**: ** *p* < 0.01 (Control vs. 10, 20, 50 and 100 µM).

The generation of ROS was evaluated to determine any relation with the cytotoxic behavior of complexes **2** and **6**. Increased levels of ROS can induce cell death by activating several signaling pathways resulting in cell apoptosis [49]. The cell-permeant 2′,7′-dichlorodihydrofluorescein diacetate (H_2_DCFDA) was used as a probe for ROS. Inside the cells, its oxidation to fluorescent 2′,7′-dichlorofluorescein (DCF) should be interpreted as an indication of the cellular oxidative stress. Essentially, peroxides can be detected using the H_2_DCFDA probe. The effect of the complexes **2** and **6** on ROS production by A2780 cells are shown in Figure 8. As can be observed the levels of ROS production upon 1 h incubation are concentration-dependent, with the complexes in the range 10–100 μM. The generation of ROS compared with the control (non-treated cells) increased ca. 3 fold at 100 μM for **6** and ca. 2 fold for **2** at the same concentration, in agreement with their cytotoxic profile. This observation is in line with the reported redox behavior, as compound **6** is the most readily reduced to the dianionic form, followed by compound **2** (Table 1).

#### 2.2.4. Apoptosis by Caspases 3/7 Activation

**Figure 9:**
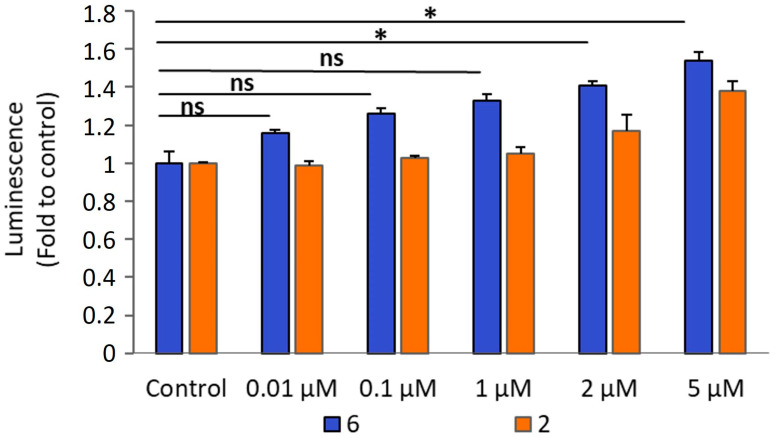
Caspase 3/7 activity in the A2780 cells after 24 h exposure to the complexes [Au(dcbdt)_2_]^−^ (**2**) and [Au(dcdmp)_2_]^−^ (**6**) at concentrations below their IC_50_ values. Results in luminescence (fold to control) are mean ± SD of at least 2 experiments with 4 replicates per condition. * *p* < 0.05, ns = not significant.

A large body of evidence indicates that gold complexes induce apoptosis in cancer cells and even in cells that are resistant to specific drugs [50]. Caspases are a family of proteases that play a crucial role in apoptosis, making them potential targets for a plethora of disease conditions such as cancer. Caspases function in a cascade of cleavage events that culminate in cell death. Caspases involved in apoptosis are initiators (caspases-8 and -9) or executioners (caspases-3, -6 and -7), which cleave selected targets, enabling cell death. We used the active form of caspase-3 and -7 for the detection of apoptotic events induced by the gold complexes **2** and **6**. As depicted in Figure 9, the ability of the complexes to activate caspase-3/7 correlated with their cytotoxicity. Complex **6** showed the highest caspase activation activity, in contrast to **2**, which we hypothesize to induce cell death by other mechanisms.

#### 2.2.5. Necrosis by Lactate Dehydrogenase (LDH) Release

Necrotic cell death can be evaluated by the detection of lactate dehydrogenase (LDH). LDH is a soluble cytoplasmic oxidoreductase that is released into the extracellular space when the plasma membrane is damaged [51,52]. To detect the leakage of LDH into cell culture medium, an assay was used based on the oxidation of lactate to pyruvate catalysed by LDH to produce NADH that convert the tetrazolium salt MTT to a formazan product. The method allows the quantification of only dead (necrotic, or late apoptotic) cells rather than cells stopping to proliferate (e.g., senescent cells), or early apoptotic cells which keep their plasma membrane intact [53].

**Figure 10:**
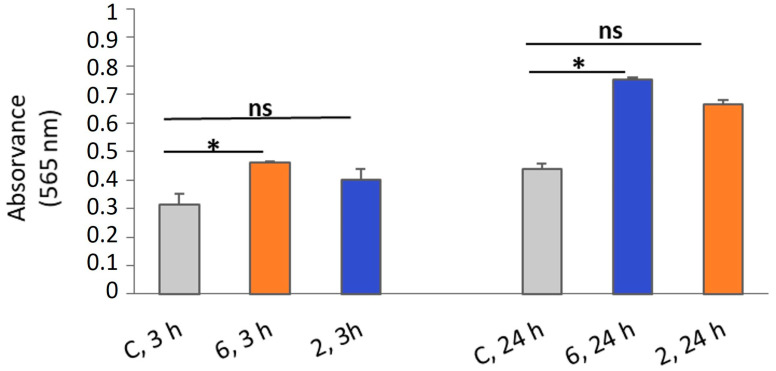
LDH release from A2780 cells monitored after 25 min. reaction time. The cells were previously exposure to complexes [Au(dcbdt)_2_]^−^ (**2**) and [Au(dcdmp)_2_]^−^ (**6**) and at 3 h and 24 incubation at concentrations corresponding to their IC_50_ values (80 µM and 20 µM, 3 h; 5 µM, 24 h) for **2** and **6**, respectively. Untreated cells (C = Control) were included for comparison. Results are expressed as absorbance units at 565 nm, from a representative experiment performed with four replicates per condition (mean ± SD). * *p* < 0.05, ns = not significant.

The LDH release by the A2780 cells was evaluated upon treatment with complexes **2** and **6** at concentrations corresponding to their IC_50_ values determined at 3 h and 24 h incubation. Compared to the respective controls, the LDH release from the cells was superior after treatment with the complexes (Figure 10). Moreover, the increase in the incubation time resulted in an increase in LDH release, indicating a deleterious effect on the cells. Although LDH release indicate a toxic response in the cells that increase with time after treatment with the complexes, it was also observed high levels in the control cells. This observation could be related with cancer cells that exhibit high LDH expression due to cancer metabolism [54]. In addition, the LDH release is not associated with the cellular uptake ratios, which could possibly be due to the fact that complexes could act through different pathways.

#### 2.2.6. Antimicrobial Activity

The antimicrobial properties of the compounds **2** and **6** were evaluated based on the determination of the minimal inhibitory concentration (MIC) values, using the microdilution method. The MIC values were determined for the Gram-positive bacteria *Staphylococcus aureus* Newman, the Gram-negative bacteria *Escherichia coli* ATCC25922 and *Burkholderia contaminans* IST408, as well as for the pathogenic yeasts *Candida glabrata* CBS138 and *Candida albicans* SC5134 (Table 3).

Among the bacterial and fungal strains tested, compound **6** presented antimicrobial activity solely against *S. aureus* Newman. In contrast, compound **2** had no antimicrobial activity against any of the tested bacterial and fungal strains for concentrations up to 62.5 or 125 µg/mL, respectively. In contrast with these results, the reference drug auranofin has shown significant antimicrobial activity, particularly against Gram-positive bacteria but has limited activity against Gram-negative bacteria due to the low permeability of their outer membrane [36].

To gain further insight into structure–activity relationships regarding antimicrobial properties, the activity of compound **6** was compared to that of its related complex, *n*-Bu_4_N[Au(qdt)_2_]. The first notable difference is that compound **6** exhibits activity exclusively against the Gram-positive bacterium *S. aureus* Newman, whereas *n*-Bu_4_N[Au(qdt)_2_] is also active against the fungal strain *C. glabrata* CBS138 [36]. Secondly, the potency of compound **6** is significantly lower than that of *n*-Bu_4_N[Au(qdt)_2_]. These observations suggest that, in this case, the presence of a pyrazine ring within an extended aromatic system plays a critical role in the antimicrobial activity. Moreover, substitution of the pyrazine ring with another aromatic unit, as in the quinoxaline core of *n*-Bu_4_N[Au(qdt)_2_], appears to be more favorable for antimicrobial efficacy than substitution with cyanonitrile electron-withdrawing groups.

## 3. Materials and Methods

### 3.1. Synthesis and Characterization of Complexes ***1***–***6***

Compounds **6** was synthesized as previously described [43]. Other chemicals, and the HPLC column, were commercially obtained and used without any further purification. Elemental analyses of the compounds were performed using an EA 110 CE Instruments automatic analyzer (Wigan, UK).

High Performance Liquid Chromatography (HPLC) analysis of the gold complexes was performed on a Perkin Elmer series 200 analytical HPLC instrument (PerkinElmer, Waltham, MA, USA) equipped with a tunable UV-Vis detector set to 300 nm. The column used was a Supelco Analytical Discovery BIOWide Pore C18-5 column (250 × 4.6 mm^2^, 300 Å pore size, 5 μm particle size) with a flow rate of 1 mL/min. HPLC solvents consisted of 0.1% CF_3_COOH in H_2_O (eluent A) and 0.1% CF_3_COOH in acetonitrile (eluent B), and the following gradient was used: 60% (A) and 40% (B) to 100% (B) in 15 min followed by 5 min at 100% (B).

High-resolution electrospray ionization (ESI) mass spectra, in positive ion mode, were obtained in a QqTOF Compact mass spectrometer (Bruker Daltonics, Billerica, MA, USA). The TOF analyzer was calibrated in the *m*/*z* range 200–2000 using a calibration standard (Tune mix solution, Agilent, Santa Clara, CA, USA). Full scan mass spectra were acquired over a range of 200–2000 *m*/*z* at a spectra rate of 1 Hz. Data was processed using Compass Data Analysis 6.1 software. The samples were collected from HPLC purification in acetonitrile and water and were diluted in acetonitrile (the final concentration was ca. 1 × 10^−6^ M) and directly injected into the spectrometer.

Tetrabutylammonium Salt of gold(III) bis(benzene−1,2-dithiolate), *n*-Bu_4_N[Au(bdt)_2_] (**1**): Benzene-1,2-dithiol (0.100 g; 0.703 mmol) was dissolved in 5 mL of NaOH aq., under inert atmosphere in a warm water bath (~45 °C) until the solution turned to a translucent yellow. Potassium tetrachloroaurate (KAuCl_4_; 0.1330 g; 0.352 mmol) was dissolved in water (5 mL), added to the reaction mixture and stirred for ~30 min. The mixture was filtered to a solution of *n*-Bu_4_NBr (0.1134 g, 0.352 mmol), also in water (2 mL), and the crude material was recrystallized from acetone/isopropanol to give **1** as green plates. Yield 51%. C_28_H_44_NAuS_4_: calcd. C 46.71, H 6.16, N 1.95, S 17.81; found C 46.89, H 6.21, N 1.94, S 17.56. HPLC Analysis retention time (Rt) = 12.30 min. MS: *m*/*z* (%): 476.9081 (100) [M]^−^.

Tetrabutylammonium Salt of gold(III) bis(4,5-dicyanobenzene-1,2-dithiolate), *n*-Bu_4_N[Au(dcbdt)_2_] (**2**): The same procedure was used as for compound **1**, using 5,6-dicyanobenzene-1,3-dithiol-2-thione instead of benzene-1,2-dithiol. Yield 43%. C_32_H_40_N_5_AuS_4_: calcd. C 46.88, H 4.92, N 8.45, S 15.64; found C 46.70, H 4.86, N 8.20, S 15.45. Rt = 17.13 min. MS: *m*/*z* (%): 576.9083 (100) [M]^−^.

Tetrabutylammonium Salt of gold(III) bis(3-cyanobenzene-1,2-dithiolate), *n*-Bu_4_N[Au(3-cbdt)_2_] (**3**): The same procedure was used as for compound **1**, using 3-cyanobenzene-1,3-dithiol-2-ketone instead of benzene-1,2-dithiol. Yield 47%. C_30_H_42_N_3_AuS_4_: calcd. C 46.80, H 5.50, N 5.46, S 16.66; found C 46.51, H 5.23, N 5.28, S 16.40. Rt = 13.12 min. MS: *m*/*z* (%): 526.9083 (100) [M]^−^.

Tetrabutylammonium Salt of gold(III) bis(4-cyanobenzene-1,2-dithiolate), *n*-Bu_4_N[Au(4-cbdt)_2_] (**4**): The same procedure was used as for compound **1**, using 4-cyanobenzene-1,3-dithiol-2-ketone instead of benzene-1,2-dithiol. Yield 63%. C_30_H_42_N_3_AuS_4_: calcd. C 46.80, H 5.50, N 5.46, S 16.66; found C 46.72, H 5.41, N 5.38, S 16.46. (Rt) = 12.30 min. MS: *m*/*z* (%): 526.9080 (100) [M]^−^.

Tetrabutylammonium Salt of gold(III) bis(pyrazina-1,2-dithiolate), *n*-Bu_4_N[Au(pdt)_2_] (**5**): The same procedure was used as for compound **1**, using pyrazine-1,2-dithiol instead of benzene-1,2-dithiol. Yield 12%. C_24_H_40_N_5_AuS_4_: calcd. C 39.82, H 5.57, N 9.68, S 17.72; found C 40.01, H 5.43, N 9.50, S 17.61. Rt = 17.24 min. MS: *m*/*z* (%): 480.8983 (100) [M]^−^.

Tetrabutylammonium Salt of gold(III) bis(4,5-dicyanopyrazine-1,2-dithiolate), *n*-Bu_4_N[Au(dcdmp)_2_] (**6**): Synthesis as in [43]. Rt = 17.24 min. MS: *m*/*z* (%): 580.8784 (100) [M]^−^.

### 3.2. Studies on Complexes Stability in Solution by UV–Vis Spectroscopy

The stability of the compounds in solution was assessed by UV-Vis spectroscopy at 37° degrees over a 24 h period, with measurements taken at 0, 3 and 24 h. All 6 compounds were initially dissolved in DMSO. From these stock solutions, two experimental conditions were established to evaluate the impact of the medium on compound stability: 100% DMSO and DMEM with 10% (*v*/*v*) FBS and 1% (*v*/*v*) DMSO. Each measurement included a baseline correction using a prepared blank that was also incubated at 37 °C, corresponding to the solvent composition of the respective condition. The measurements were made using an Agilent Technologies Cary 60 UV-vis Spectrophotometer (Santa Clara, CA, USA).

### 3.3. Cytotoxic Activity Against Ovarian Cancer

The cytotoxic activity of the complexes was evaluated in cisplatin-sensitive (A2780) and cisplatin-resistant (A2780cisR) ovarian cancer cells (Sigma-Aldrich, St. Louis, MO, USA). Cells were grown in RPMI-1640 medium (Gibco, Thermo Fisher, Waltham, MA, USA) supplemented with 10% fetal bovine serum (FBS). Both cell lines were cultured at 37 °C, 5% CO_2_ in a humidified atmosphere.

For the assays, cells were seeded in 96-well plates at a density of 2 × 10^4^ cells in 200 μL medium and allowed to adhere overnight. The bis (dithiolene) complexes were initially dissolved in DMSO to prepare a 10 mM stock solution and then in culture medium to prepare serial dilutions in the range 0.01–50 µM. At several incubation times, the cellular viability was measured by the colorimetric MTT assay, as previously described [36].

### 3.4. Cellular Uptake

#### 3.4.1. Cellular Uptake by PIXE

A2780 cells were incubated with the complexes **2** and **6** during 3 h at 50 and 20 μM, respectively. Briefly, freeze-dried cell pellets were subjected to microwave-assisted acid digestion using a 1:3 molar ratio of nitric and hydrochloric acids, respectively, together with yttrium (100 mg/L) as the internal standard, as previously described [55]. The Au was measured using particle-induced X-ray emission (PIXE), and concentrations were expressed in ng Au/10^6^ cells.

#### 3.4.2. Cellular Uptake by ICP-MS

A2780 cells were incubated with the complexes **2** and **6** during 3 h at 50 and 20 μM, respectively, and 24 h at 5 μM. The cell pellets were digested with nitric acid at 100 °C for 12 h and the solution diluted to 10 mL. The Au content in each cell fraction was measured using a Thermo X-Series Quadrupole ICP-MS (Thermo Fisher Scientific Inc., Waltham, MA, USA), and the Au contents were calculated and expressed in ng/10^6^ cells.

### 3.5. Intracellular ROS by H2DCF-DA

Detection of ROS, mainly peroxides were measured using the 2′,7′- diacetate (H_2_DCF-DA) fluorescent probe (Invitrogen, Thermo Fisher Scientific, Waltham, MA, USA). The assay is based on the cleavage of H_2_DCF-DA by intracellular esterase enzymes which are oxidized by ROS to DCF. Complexes **2** and **6** were assayed at 10, 20, 50 and 100 μM for 1 h, following a method previously described [36]. The DCF green fluorescence (λ_ex_ = 485 nm; λ_em_ = 530 nm) was measured using a Varioskan LUX scanning multimode reader (Thermo Fisher Scientific). Results (mean ± SD) were expressed as the fold change in fluorescence levels compared to controls (non-treated cells).

### 3.6. Apoptosis (Caspase 3/7)

Caspase-3 and -7 activities were assessed using a Caspase-Glo^®^3/7 assay (Promega, Madison, WI, USA) as previously described [36]. The assays were carried out with the A2780 cells in white-walled 96 well plates, treated with the complexes at concentrations below their IC_50_ values for 24 h. After incubation, the Caspase 3/7^®^ reagent was added, resulting in cell lysis, followed by caspase cleavage of the substrate and generation of a luminescent signal, which is proportional to the amount of caspase activity. The luminescence intensity was measured using a Varioskan LUX scanning multimode reader (Thermo Fisher Scientific). Results (mean ± SD) were expressed as the fold change in luminescence compared with controls (untreated cells).

### 3.7. Lactate Dehydrogenase Release

This method is based on the release of cytosolic glycolytic enzyme lactate dehydrogenase (LDH) into the medium due to plasma membrane rupture due to cell death. LDH released in culture supernatants is then quantified by an enzymatic assay (Sigma-Aldrich) after 25 min reaction following the recommended procedure for cell lysates. Briefly, after treatment with the complexes **2** and **6**, supernatant and cells were collected and homogenized by sonication. Samples were centrifuged at 10,000× *g* for 15 min at 4 °C. The supernatant of each condition was removed and stored at −20 °C until use. The assay is based in the conversion of a tetrazolium salt (MTT) into a red formazan product. The intensity of color is proportional to the LDH released, and absorbance data at 565 nm were collected using a standard 96-well plate reader.

### 3.8. Antimicrobial Activity Determination

Minimum Inhibitory concentrations (MIC) were determined for the bacterial strains *Staphylococcus aureus* Newman, *Escherichia coli* ATCC25922, *Burkholderia contaminans* IST408, and the fungal strains *Candida glabrata* CBS138 and *Candida albicans* SC5134. Bacterial and fungal strains were maintained in Lennox Broth (LB) and Yeast Extract–Peptone–Dextrose (YPD) solid medium, respectively [36,37].

Compounds **2** and **6** stock solutions were prepared in 100% DMSO, at final concentrations of 5 mg/mL. The antibacterial susceptibility of the compounds was assessed using standard methods recommended by the European Committee on Antimicrobial Susceptibility Testing (EUCAST) for non-fastidious bacteria [56]. Final compounds concentrations tested ranged 125 µg/mL to 0.49 µg/mL, contained in the wells of 96-well microplates, in a total volume of 200 µL per well. An initial 5 × 10^5^ CFU/mL were used for the tested bacteria. After incubation for 22 h at 37 °C, the optical density of cultures was measured at a wavelength of 640 nm as previously described [37,38].

The EUCAST microdilution method recommended for *Candida* species MIC determination [57] was used, using compounds with concentrations ranging from 62.5 µg/mL to 0.24 µg/mL, and following previously published procedures [36,37]. After incubation for 22 h at 30 °C, cultures optical density was measured at a wavelength of 530 nm as previously described [36,37].

A minimum of three independent experiments carried out in duplicate were performed for each compound testes. MIC values were estimated using a modified Gompertz equation with GraphPad Prism software (version 6.07) [58]. In each experiment, positive (no compound) and negative controls (no microorganism) were included. Auranofin was also used in bacterial and fungal MIC determinations as a reference antimicrobial, as described in [37]. Additionally, the effect of 5% (*v*/*v*) or 2.5% (*v*/*v*) DMSO on bacterial or fungal growth, respectively, were assessed, and no effects were observed.

## 4. Conclusions

In this study, we investigated six monoanionic gold (bis)dithiolate complexes: [Au(bdt)_2_]^−^ (**1**), [Au(dcbdt)_2_]^−^ (**2**), [Au(3-cbdt)_2_]^−^ (**3**), [Au(4-cbdt)_2_]^−^ (**4**), [Au(pdt)_2_]^−^ (**5**) and [Au(dcdmp)_2_]^−^ (**6**), for their biological activity against ovarian cancer cell lines (A2780 and A2780cisR, Gram-positive bacteria (*Staphylococcus aureus* Newman), Gram-negative bacteria (*Escherichia coli* ATCC25922 and *Burkholderia contaminans* IST408), and pathogenic yeasts (*Candida glabrata* CBS138 and *Candida albicans* SC5134). The structural similarities among these complexes facilitated an examination of how subtle molecular modifications influence their biological activity. Our findings indicate that the cytotoxic profiles of these compounds are significantly influenced by the presence and quantity of cyanonitrile groups within the cyclic aromatic system. Notably, complexes **2** and **6**, both containing two cyanonitrile groups, exhibited the highest levels of activity, demonstrating after 24 h treatment comparable cytotoxic effects against A2780 ovarian cancer cells (IC_50_ ~ 5 µM). Furthermore, both complexes displayed similar anticancer activity in the A2780cisR cells, suggesting their potential to overcome cisplatin resistance. Importantly, the observed cytotoxicity was not correlated with their cellular uptake ability. Both complexes were found to induce the formation of reactive oxygen species (ROS), activate caspase−3/7, and promote necrosis, as evidenced by lactate dehydrogenase (LDH) release, in a dose-dependent manner, with complex **6** exhibiting a more pronounced effect than complex **2**. Among the tested bacterial and fungal strains, only complex **6** demonstrated antimicrobial activity against *S. aureus* Newman.

A clear SAR was observed at 48 h, with cyanonitrile substituents enhancing anticancer activity in a position- and number-dependent manner. Phenyl-based compounds, particularly the doubly substituted compound **2**, exhibited the highest activity, highlighting the role of electron-withdrawing groups. Compared to the related complex *n*-Bu_4_N[Au(qdt)_2_], compound **6** displayed narrower antimicrobial activity, being active only against S. aureus, and with reduced potency. These findings indicate that extended aromatic systems, such as the quinoxaline core, are more favorable for antimicrobial efficacy than cyanonitrile substitution on a pyrazine ring. Still, these results suggest that complex **6** holds promise as a novel lead compound for both anticancer and antibacterial applications.

## Figures and Tables

**Table 1:** Redox potentials for compounds **1**–**6**.

Compound	E_1/2_ (mV) [M(L)_2_]^0^/[M(L)_2_]^+^	E_1/2_ (mV) [M(L)_2_]^−^/[M(L)_2_]^0^	E_1/2_ (mV) [M(L)_2_]^−^/[M(L)_5_]^−2^	E_1/2_ (mV) [M(L)_2_]^−2^/[Au(L)_2_]^−^	Ref.
**1** ^(a)^	_	365	_	_	[40]
**2** ^(b)^	_	+1200 *	+400	−1330	[45]
**3** ^(c)^	_	+530	_	_	[44]
**4** ^(d)^	+1410	+490	_	−1520	[42]
**5** ^(e)^	+1500 *	+720 *	_	−1665	[41]
**6** ^(f)^	_	_	_	−629	[43]

* Only the oxidation process is observed. ^(a)^ The solvent used was DMF. The potentials are refered relatively to Fc/Fc+ couple; ^(b)^ the solvent used was dichloromethane. Ag^+^/AgNO_3_ (10^−3^ M, acetonitrile solution) reference electrode. No internal reference was used; ^(c)^ the solvent used was acetonitrile. Ag^+^/AgNO_3_ (10^−3^ M, acetonitrile solution). No internal reference was used; ^(d)^ solvent used was acetonitrile. Ag^+^/AgNO_3_ (10^−3^ M, acetonitrile solution). No internal reference was used; ^(e)^ the solvent used was acetonitrile. The potentials are refered relatively to Fc/Fc+ couple; ^(f)^ the solvent used was acetonitrile. Ag^+^/AgNO_3_ (10^−3^ M, acetonitrile solution). No internal reference was used.

**Table 2:** The IC_50_ values (µM) found for complexes (**1**–**6**) in the ovarian cancer cells A2780 and A2780cisR at several time points. Results are the mean ± SD of two independent experiments performed with six replicates per condition. * Data from Ref. [36].

Compound	A2780			A2780cisR	
	3 h	24 h	48 h	24 h	48 h
[Au(bdt)_2_]^−^ (**1**)	>100	31.6 ± 4.2	12.1 ± 2.2	51.4 ± 6.4	48.7 ± 10
[Au(dcbdt)_2_]^−^ (**2**)	80.6 ± 16	5.20 ± 1.8	0.11 ± 0.04	5.62 ± 2.1	1.81 ± 0.7
[Au(3-cbdt)_2_]^−^ (**3**)	>100	8.9 ± 2.0	2.61 ± 1.3	35.3 ± 4.6	18.9 ± 3.6
[Au(4-cbdt)_2_]^−^ (**4**)	>100	17.4 ± 3.6	5.89 ± 1.3	13.5 ± 2.2	7.13 ± 1.0
[Au(pdt)_2_]^−^ (**5**)	65.3 ± 17	30.9 ± 4.4	13.6 ± 4.2	59.5 ± 6.0	53.5 ± 11
[Au(dcdmp)_2_]^−^ (**6**)	21.8 ± 5.3	5.10 ± 0.9	3.77 ± 0.9	3.90 ± 1.0	11.7 ± 2.8
Cisplatin	……	21.1 ± 5.0 *	3.6 ± 1.3 *	45.0 ± 18	35.8 ± 13 *

**Table 3:** Minimal inhibitory concentration (MIC) for the compounds [Au(dcbdt)_2_]^−^ (**2**) and [Au(dcdmp)_2_]^−^ (**6**), towards the indicated bacterial and fungal strains. Results are presented as the means ± SD of at least 3 experiments carried out in duplicate. Auranofin was used as control as described in ref. [36].

	MIC (µg/mL)	
	2	6
*S. aureus* Newman	>125	60 ± 4.3
*E. coli* ATCC25922	>125	>125
*B. contaminans* IST408	>125	>125
*C. glabrata* CBS138	>62.5	>62.5
*C. albicans* SC5134	>62.5	>62.5

## Data Availability

The original contributions presented in this study are included in the article/Supplementary Materials. Further inquiries can be directed to the corresponding authors.

## Supplementary Materials

The following supporting information can be downloaded at: https://www.mdpi.com/article/10.3390/molecules30153270/s1, Figure S1: UV—Vis spectra of complex 1 (a) and 3 (c) in DMSO solution, and in phenol red-free DMEM/F12 medium in the presence of FBS (b) and (d), for 1 and 3 respectively, at 0 h (t0, green), 3 h (t3, yellow) and 24 h (t24, pink); Figure S2: UV—Vis spectra of complex 4 (a) and 5 (c) in DMSO solution, and in phenol red-free DMEM/F12 medium in the presence of FBS (b) and (d), for 4 and 5 respectively, at 0 h (t0, green), 3 h (t3, yellow) and 24 h (t24, pink).
