# Supplementary material for: Gold(III) Complexes with Aromatic Cyano-Substituted Bisdithiolate Ligands as Potential Anticancer and Antimicrobial Agents
Source: Molecules. 2025 Aug 4;30(15):3270. doi: 10.3390/molecules30153270 (PMC12348044; doi:10.3390/molecules30153270)
Supplement: Supplementary file 1 [file molecules-30-03270-s001.zip › molecules-3712217-supplementary.pdf]

Gold(III) Complexes with Aromatic Cyano-Substituted Bisdithiolate Ligands as Potential Anticancer and Antimicrobial Agents

Dulce Belo [1,2,*], Sandra Rabaça [1,2], Sara G. Fava [1], Sílvia A. Sousa [3,4], Diogo Coelho [3,4], Jorge H. Leitão [3,4], Teresa Pinheiro [2,3], Célia Fernandes [1,2] and Fernanda Marques [1,2,*]

[1] Centro de Ciências e Tecnologias Nucleares, Instituto Superior Técnico, Universidade de Lisboa, Estrada Nacional 10, Km 139.7, Bobadela, 2695-066 Loures, Portugal; sandrar@ctn.tecnico.ulisboa.pt (S.R.); sara.f.goncalves@tecnico.ulisboa.pt (S.G.F.); celiaf@ctn.tecnico.ulisboa.pt (C.F.)

[2] Departamento de Engenharia e Ciências Nucleares, Instituto Superior Técnico, Universidade de Lisboa, Estrada Nacional 10, Km 139.7, Bobadela, 2695-066 Loures, Portugal; murmur@ctn.tecnico.ulisboa.pt

[3] iBB-Institute for Bioengineering and Biosciences, Associate Laboratory i4HB—Institute for Health and Bioeconomy, Instituto Superior Técnico, Universidade de Lisboa, Av. Rovisco Pais, 1049-001 Lisboa, Portugal; sousasilvia@tecnico.ulisboa.pt (S.A.S.); diogocoelho@tecnico.ulisboa.pt (D.C.); jorgeleitao@tecnico.ulisboa.pt (J.H.L.)

[4] Department of Bioengineering, Instituto Superior Técnico, Universidade de Lisboa, 1049-001 Lisboa, Portugal

* Correspondence: dbelo@ctn.tecnico.ulisboa.pt (D.B.); fmarujo@ctn.tecnico.ulisboa.pt (F.M.)

Studies on complexes stability in solution by UV–vis spectroscopy

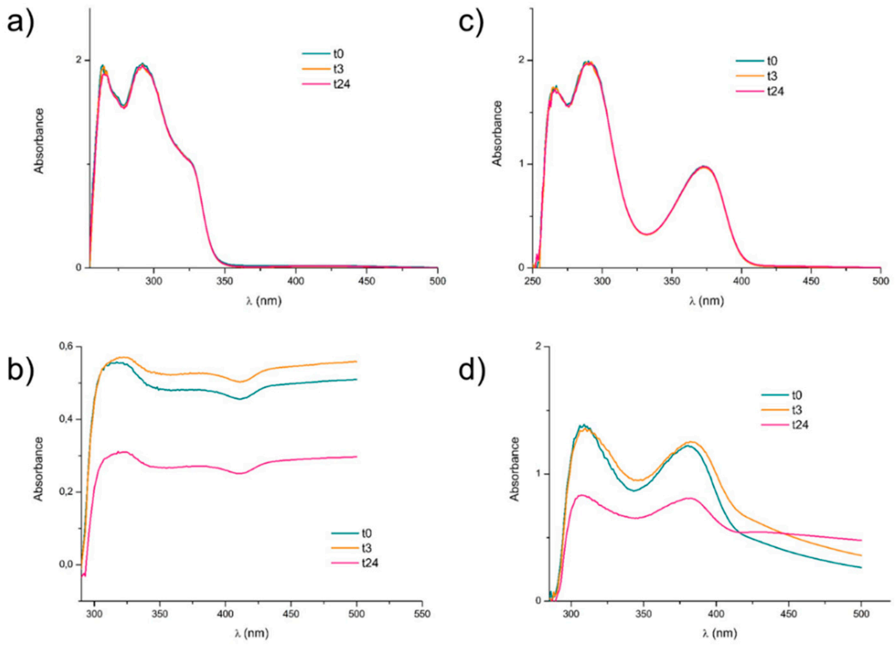

Figure S1. UV–Vis spectra of complex **1** (a) and **3** (c) in DMSO solution, and in phenol red-free DMEM/F12 medium in the presence of FBS b) and d), for **1** and **3** respectively, at 0 h (t0, green), 3 h (t3, yellow) and 24 h (t24, pink).

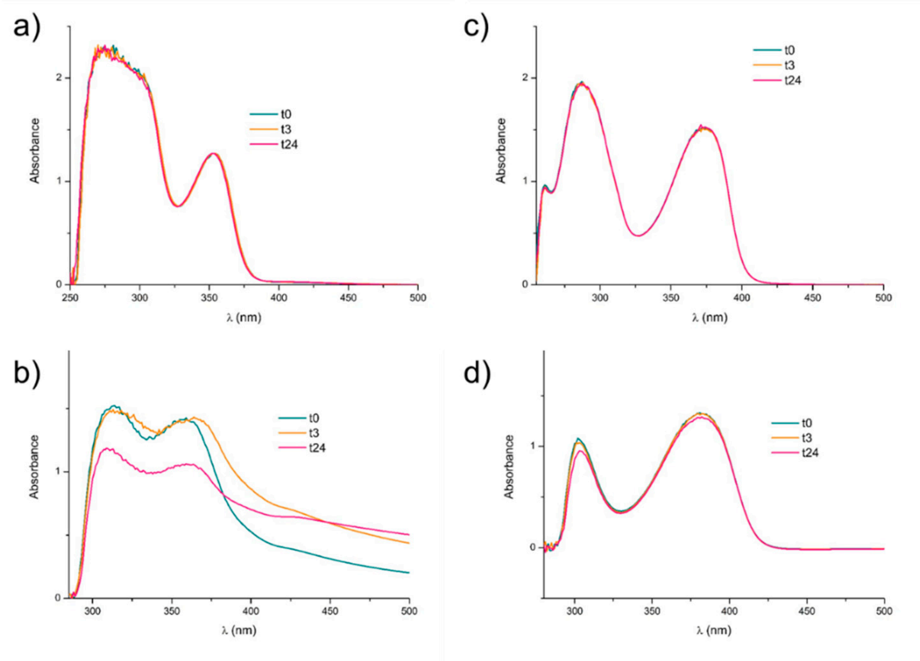

Figure S2. UV–Vis spectra of complex **4** (a) and **5** (c) in DMSO solution, and in phenol red-free DMEM/F12 medium in the presence of FBS b) and d), for **4** and **5** respectively, at 0 h (t0, green), 3 h (t3, yellow) and 24 h (t24, pink).
